# McArdle’s disease presents with multiple large vessel lesions

**DOI:** 10.1515/rir-2024-0032

**Published:** 2025-01-09

**Authors:** Shuning Guo, Yanping Wei, Wu Di, Jing Li

**Affiliations:** Department of Rheumatology and Clinical Immunology, Peking Union Medical College Hospital, Chinese Academy of Medical Sciences, Peking Union Medical College, Beijing, China; National Clinical Research Center for Dermatologic and Immunologic Diseases (NCRC-DID), Ministry of Science & Technology, Beijing, China; State Key Laboratory of Complex Severe and Rare Diseases, Peking Union Medical College Hospital, Beijing, China; Key Laboratory of Rheumatology and Clinical Immunology, Ministry of Education, Beijing, China; Department of Neurology, Peking Union Medical College Hospital, Chinese Academy of Medical Sciences and Peking Union Medical College, Beijing, China

Dear Editor,

A 35-year-old woman was admitted to the hospital one week before delivery due to sudden severe headache accompanied by blurred vision, subsequently diagnosed as severe preeclampsia. During her follow-up visit six months post-delivery, physical examination revealed bilateral carotid arteries, the right upper limb artery, and both lower limb arteries were found to be pulseless. Vascular murmurs were detected in bilateral carotid arteries and subclavian arteries. Ultrasonography disclosed severe stenosis of the bilateral common carotid arteries and the external carotid arteries, as well as uneven thickness and localized dilation of the bilateral iliac arteries. Besides, laboratory tests indicated persistently elevated serum creatine kinase (CK) levels at rest, with significant fluctuations during monitoring (ranging from 1209 U/L to 4419 U/L), despite the absence of notable patient discomfort.

The patient was diagnosed with Takayasu’s arteritis (TAK) and underwent surgery for carotid artery intimal dissection. Following the procedure, the patient experienced a transient loss of consciousness and headache. The computed tomography scan showed subarachnoid hemorrhage in the right frontal lobe (Figure 1). Additionally, the patient suffered an epileptic seizure. Upon discharge, the patient was prescribed aspirin, levetiracetam, prednisolone and methotrexate.

**Figure 1:**
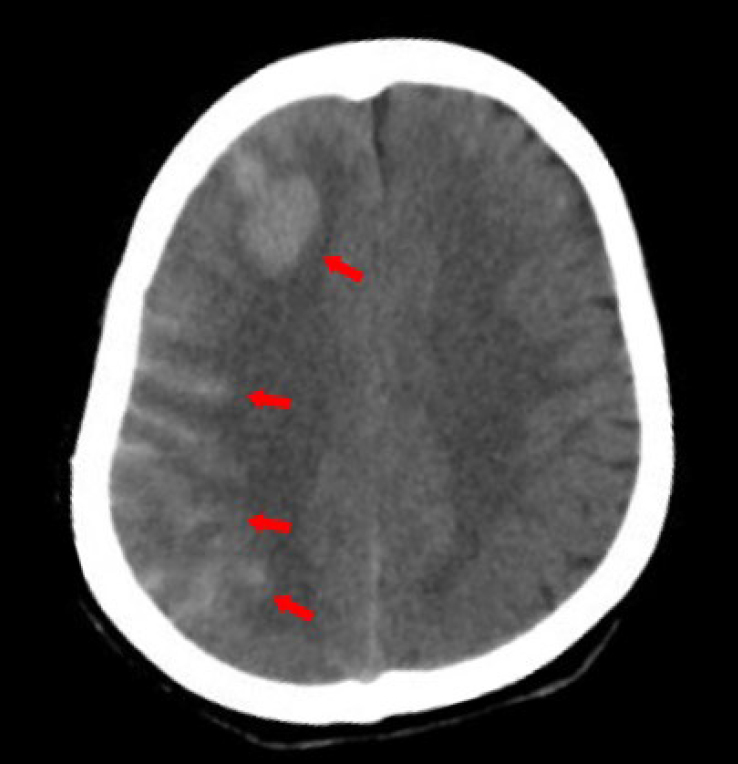
Computed tomography evidence of intracranial hemorrhage after surgery.

During the follow-up, cardiac ultrasound disclosed aortic valve thickening and mild aortic regurgitation. Subsequent routine ultrasound examinations that were performed after 18 months identified a true aneurysm of the distal abdominal aorta. Besides, the CK level remained consistently elevated and showed no response to immunosuppressive therapy. During the consultation with a neurologist, the patient recalled relatively poor endurance in sports since childhood alongside unrestricted daily activities. Furthermore, she experienced lower limb weakness and cramps when climbing and running. No similar manifestations were noted in her family members. The molecular genetic testing of peripheral blood showed the c. 148C > T mutation in the PYGM gene, leading to a diagnosis of McArdle’s disease. The patient was advised to abstain from intense exercise to reduce the symptoms of myalgia and the risk of rhabdomyolysis.

McArdle’s disease, known as glycogen storage disease V, arises from mutations in the PYGM gene encoding muscle glycogen phosphorylase (myophosphorylase), resulting in exercise intolerance including fatigue, muscle soreness, and cramps during the initial stages of exercise.^[1]^ The onset of symptoms typically occurs during childhood, with approximately 60% in the first decade and 28% in the second decade of life. However, the condition is often diagnosed late in adulthood and frequently misdiagnosed.^[2,3]^ TAK is characterized by granulomatous inflammation of the aorta and its major branches, and manifested as systemic symptoms, vascular lesions including wall thickening, luminal stenosis or occlusion, aneurysm formation and ischemic manifestations within affected organs. TAK primarily impacts females, typically occurring between the ages of 10 and 40 years.^[4]^ Generally, McArdle’s disease primarily affects skeletal muscle with infrequent involvement of cardiac and vascular smooth muscles. Conversely, TAK typically leads to vascular smooth muscle dysfunction. This report describes a rare case of a 35-year-old woman diagnosed with McArdle’s disease combined with Takayasu’s arteritis who presented with muti-vascular lesions. The involvement of cardiovascular system in McArdle’s disease is quite rare, and the coexistence of McArdle’s disease and Takayasu’s arteritis seems to jointly exacerbate the muscle damage.

Intracranial hemorrhage is quite rare in patients with TAK. Besides, this is also the first report in the literature that illustrates McArdle’s disease combined with aneurysm and intracranial hemorrhage. The involvement of cardiovascular system in McArdle’s disease is notably rare. Previous studies have documented hypertrophic cardiomyopathy and electro-cardiogram changes in McArdle’s disease ^[5,6]^ Pompe’s disease, attributed to a deficiency in acid α-glucosidase, is referred as type II of glycogen storage disease, and manifests as hypertrophic cardiomyopathy, hypotonia and motor delay. Existing reports suggest a prevalence of intracranial artery abnormalities in Pompe’s disease, with cerebrovascular disease posing a potential risk factor for mortality in late-onset Pompe’s disease patients.^[7]^ These data demonstrate that although not common, the degenerative vascular disorders may exist in glycogen storage diseases. McArdle’s disease seems to increase the potential risk of cerebral hemorrhage which may pose surgical challenges in the future. Due to weakened or degenerated status of blood vessel walls, this patient was more prone to developing aneurysm and valve damage.

Another noteworthy point is that the patient developed an aneurysm, indicating the dysfunction of vascular smooth muscle cells. The prevailing hypothesis in TAK is that vascular smooth muscle cells may function as local enhancers of the inflammatory processes within vascular lesions. A substantial number of patients with TAK exhibited aneurysmal diseases.^[8]^ This evidence supports the relationship between TAK and aneurysm in our case. McArdle’s disease has a specific preference for skeletal muscle. Although no prior reports documented the involvement of vascular smooth muscle in McArdle’s disease, the biopsy of a Pompe’s disease patient with thoracic aortic aneurysm showed lysosomal glycogen deposits within smooth muscle tissues, and disruption of the aortic wall architecture, characterized by excessive fragmentation of elastic fibers within the media.^[9]^ We speculate that the aneurysm in our case may be caused or aggravated by McArdle’s disease leading to glycogen accumulation and functional degradation in the vascular smooth muscle cells, which requires confirmation of myophosphorylase activity in vascular smooth muscle *via* biopsy. Regardless of the cause of the aneurysm, aneurysms carry a risk of rupture and are associated with a higher rate of relapse in TAK, which requires clinicians to pay closer attention to subsequent progression.

## References

[1] Hannah WB, Derks TGJ, Drumm ML (2023). Glycogen storage diseases. Nat Rev Dis Primers.

[2] Vieitez I, Teijeira S, Fernandez JM (2011). Molecular and clinical study of McArdle’s disease in a cohort of 123 European patients. Identification of 20 novel mutations. Neuromuscul Disord.

[3] Lucia A, Ruiz JR, Santalla A (2012). Genotypic and phenotypic features of McArdle disease: insights from the Spanish national registry. J Neurol Neurosurg Psychiatry.

[4] Pugh D, Karabayas M, Basu N (2022). Large-vessel vasculitis. Nat Rev Dis Primers.

[5] Nicholls DP, Campbell NP, Stevenson HP (1996). Angina in McArdle’s disease. Heart.

[6] Moustafa S, Patton DJ, Connelly MS. (2013). Unforeseen cardiac involvement in McArdle’s disease. Heart Lung Circ..

[7] Garibaldi M, Sacconi S, Antonini G (2017). Long term follow-up of cerebrovascular abnormalities in late onset Pompe disease (LOPD). J Neurol..

[8] Lefebvre F, Ross C, Soowamber M (2024). Aneurysmal Disease in Patients With Takayasu Arteritis. J Rheumatol..

[9] Goeber V, Banz Y, Kaeberich A (2013). Huge aneurysm of the ascending aorta in a patient with adult-type Pompe’s disease: histological findings mimicking fibrillinopathy. Eur J Cardiothorac Surg..

